# Increased Prevalence of Multidrug-Resistant *Escherichia coli* as an Adverse Effect of Excessive Antibiotic Use in a Tertiary Care Hospital in Serbia

**DOI:** 10.3390/life16091392

**Published:** 2026-08-24

**Authors:** Vladimir Zivanovic, Teodora Vitorovic, Dejan Stojakov, Biljana Carevic, Ana Bukarica, Ilija Doknic, Ljiljana Gojkovic Bukarica

**Affiliations:** 1 Department of General Surgery, University Hospital Centre “Dr Dragisa Misovic–Dedinje”, 11040 Belgrade, Serbia; teodora.vitorovic@zastitazdravlja.rs (T.V.); dejan.stojakov@med.bg.ac.rs (D.S.); 2 Department of General Surgery with Anesthesiology, Faculty of Medicine, University of Belgrade, 11000 Belgrade, Serbia; 3 Special Advisor for Infection Prevention and Control, Oslo University Hospital, N-0424 Oslo, Norway; bilcar@ous-hf.no; 4 Institute for Cardiovascular Diseases, Dedinje, University of Belgrade, 11040 Belgrade, Serbia; ana.bukarica@institutdedinje.org; 5 Faculty of Organizational Sciences, University of Belgrade, 11010 Belgrade, Serbia; doknicilija98@gmail.com; 6 Institute of Pharmacology, Clinical Pharmacology and Toxicology, Faculty of Medicine, University of Belgrade, 11000 Belgrade, Serbia; ljiljana.gojkovic-bukarica@med.bg.ac.rs

**Keywords:** anti-bacterial agents, adverse effect, bacterial resistance, infection control, cross infection, drug resistance, hospitals

## Abstract

One of the most important adverse consequences of antibiotic use is the development of bacterial resistance. This study investigated the prevalence and antimicrobial resistance patterns of multidrug-resistant (MDR) *Escherichia coli* in a tertiary care hospital during 2013–2015 and 2024, together with trends in antibiotic consumption and the molecular characteristics of resistance genes in 2024 isolates. Identification and susceptibility testing were performed using the Vitek^®^ 2 system, antibiotic consumption was assessed according to WHO ATC/DDD methodology, and resistance genes were detected by PCR. No significant differences were observed in the total number of isolates, patients, or the proportion of *E. coli* isolates during 2013–2015. However, the isolation rate of MDR *E. coli* significantly declined from 36.9% to 30%. Total antibiotic consumption remained stable, with no correlation between consumption and MDR isolation rates, although ampicillin resistance increased significantly. In 2024, MDR *E. coli* accounted for 24.3% of isolates despite lower antibiotic consumption. Compared with 2015, resistance significantly increased to amoxicillin–clavulanic acid, cefotaxime, cefepime, ceftazidime, ciprofloxacin, and levofloxacin, while ceftriaxone resistance decreased. The blaCTX-M gene was detected in 53% of bloodstream isolates, indicating widespread dissemination of ESBL-producing *E. coli* and highlighting the need for continuous surveillance and antimicrobial stewardship.

## 1. Introduction

The development of bacterial resistance is considered one of the most important adverse effects of antibiotic use. The nonrational use of antibiotics refers to using antibiotics in ways that are unnecessary, incorrect, or inappropriate. This is one of the leading causes of antibiotic resistance in pathogens, a major global public health problem [1,2].

Healthcare-associated infections (HAIs) pose an increasingly serious challenge, primarily due to their frequent association with multidrug-resistant (MDR) pathogens that typically do not respond to standard antimicrobial treatments. This leads to prolonged illness and a heightened risk of mortality [2]. In Serbia, the distribution of pathogens responsible for HAIs differs notably from that in most EU nations, where *E. coli* is recognized as the leading cause of HAIs [3]. Research conducted in Serbia by Cirkovic et al. indicates that *Klebsiella* spp. was the most commonly isolated pathogen, accounting for 16.7% of cases. Furthermore, *Acinetobacter* spp. and *P. aeruginosa* ranked as the second (15.2%) and third (10.8%) most prevalent pathogens, respectively [4]. This particular distribution of pathogens is comparable only to that of Greece and Romania, as both countries have reported similar trends in recent studies [5].

According to the 2023 annual report from the Central Asian and Eastern European Surveillance of Antimicrobial Resistance (CAESAR) network, carbapenem-resistant *E. coli* has become a significant issue in Serbia, among 30 EU countries and several nations from the former Yugoslavia. The CDC report indicates that in 2023, 10.7% of the MDR *E. coli* isolates from tested samples were reported to the National Health Safety Network. This reflects a 70% increase over the past decade (2011–2020) [6,7]. Research conducted at another Serbian hospital reveals that MDR *E. coli* isolates are showing an increasing trend in susceptibility to ceftriaxone, while a decline in susceptibility to ciprofloxacin and levofloxacin has been noted [8].

This study aimed to evaluate the prevalence and antimicrobial resistance patterns of MDR *E. coli* in a tertiary care hospital in Serbia over two study periods (2013–2015 and 2024). Specifically, we sought to (i) assess temporal changes in the prevalence of MDR *E. coli*, (ii) analyze trends in hospital antibiotic consumption (iii) examine the association between antibiotic utilization and the occurrence of MDR *E. coli*, (iv) compare antimicrobial resistance profiles between the study periods, and (v) characterize the molecular epidemiology of resistance by detecting blaCTX-M resistance gene in isolates collected in 2024. These recent data provide insight into current resistance patterns and the detection of clinically important resistance genes, although direct comparisons with the earlier period are limited by methodological differences, including changes in laboratory practice and the post-pandemic context.

## 2. Materials and Methods

### 2.1. Study Design and Hospital Setting

This retrospective observational study was conducted at the University Hospital Center “Dr Dragisa Misovic-Dedinje” in Belgrade, Serbia. Two time periods were analyzed: 2013–2015 and 2024. The 2013–2015 period was selected as a historical baseline prior to the full implementation of local antimicrobial stewardship and infection prevention measures, whereas 2024 was selected as the most recent period after the introduction of molecular diagnostic techniques (PCR) into routine practice. The long interval between the two periods reflects the availability of comparable, systematically collected microbiological and consumption data rather than a planned interruption in surveillance.

The hospital has a total of 387 beds and includes departments of laparoscopic abdominal surgery, urology, geriatrics, pulmonology, hematology, endocrinology, noninvasive cardiology, gastroenterology, and an intensive care unit (ICU). Departments of psychiatry, neurology, pediatrics, and gynecology and obstetrics were not included.

Infection prevention and control measures have been in place since 2010, aligned with international standards. An antimicrobial stewardship program was initiated in 2012, including local guidelines, restriction of selected antibiotics, surveillance of MDR organisms, and staff education. Between 2015 and 2024, additional changes in staffing, infrastructure, diagnostic practices, and infection control policies were made, including adjustments related to the COVID-19 pandemic. These contextual changes are acknowledged as potential confounders in the interpretation of temporal trends.

### 2.2. Microbiological Analysis

Clinical specimens (including blood, urine, intra-abdominal samples, wound swabs, and others) were obtained from hospitalized patients as part of standard clinical care. Only diagnostic samples were included, while surveillance cultures were excluded [9]. Duplicate isolates of the same species from the same patient within the same infectious episode were removed to avoid overrepresentation.

Bacterial identification and antimicrobial susceptibility testing were performed using the Vitek^®^ 2 system (bioMérieux, Marcy l’Etoile, France), complemented by disk diffusion and microdilution methods where appropriate. Results were interpreted according to the European Committee on Antimicrobial Susceptibility Testing (EUCAST) criteria [10,11].

Multidrug resistance (MDR) was defined as non-susceptibility to at least one agent in three or more antimicrobial categories [5]. The following antibiotics were included in susceptibility testing: ampicillin, amoxicillin–clavulanic acid, cefotaxime, ceftazidime, ceftriaxone, cefepime, ciprofloxacin, levofloxacin, gentamicin, amikacin, imipenem, meropenem, nitrofurantoin, and trimethoprim–sulfamethoxazole. Several agents became available only in 2024 (tigecycline, fosfomycin, ceftazidim/avibactam and ceftalozane/tazobaktam). Consequently, the composition of tested agents and the operationalization of MDR were not fully identical across periods, which should be considered when interpreting temporal changes in MDR prevalence.

### 2.3. Antimicrobial Consumption

Antibiotic consumption data were obtained from the hospital information system (HELIANT, Belgrade, Serbia) and classified according to the World Health Organization Anatomical Therapeutic Chemical (WHO ATC) system. Consumption was expressed as defined daily doses per 100 bed-days (DDD/100 bed-days), calculated as

DDD/100 bed-days = (total antibiotic consumption in grams × 100)/(DDD × number of bed-days)

Bed-days were calculated from the total number of beds and the bed occupancy rate. All systemic antibiotics administered to hospitalized patients were included. Some antibiotics were excluded in the early period due to market availability. Prescription of selected broad-spectrum antibiotics (e.g., carbapenems, piperacillin/tazobactam, linezolid) required approval from the institutional antimicrobial stewardship committee throughout the study. This information is provided to contextualize consumption patterns and potential changes in prescribing behavior.

### 2.4. Molecular Analysis (PCR)

In 2024, molecular detection of resistance genes was performed using the BIOFIRE^®^ FILMARRAY^®^ TORCH system (bioMérieux, Marcy l’Etoile, France) with the BIOFIRE^®^ Blood Culture Identification 2 (BCID2) Panel (bioMérieux, Marcy l’Etoile, France). This assay was applied exclusively to positive blood culture samples and detected blaCTX-M, carbapenemases (KPC, NDM, VIM, IMP, OXA-48-like), colistin resistance (mcr-1), methicillin resistance (mecA/C), and vancomycin resistance (vanA/B). Blood cultures were processed according to the manufacturer’s instructions.

Due to the introduction of PCR diagnostics in 2024, results from this year were analyzed separately and are not directly comparable with data from the earlier period. The absence of comprehensive genomic sequencing or molecular typing is an important limitation, as the available data cannot determine whether observed resistance patterns reflect clonal dissemination, repeated independent acquisition of resistance determinants, or introduction of distinct *E. coli* lineages.

### 2.5. Ethical Considerations

The study was approved by the Ethics Committee of the University Hospital Center “Dr Dragisa Misovic-Dedinje” (Belgrade, Serbia; Approval No. 29/XI-11, dated 09 November 2015). The requirement for informed consent was waived due to the retrospective nature of the study and the use of anonymized data.

### 2.6. Software

Data analysis was conducted using WHONET 5.6 software. Statistical evaluation was performed with IBM SPSS Statistics version 24 (IBM Corp., Armonk, NY, USA). Results are presented as frequencies (percentages) and means ± standard deviation (SD). Differences between groups were assessed using the independent samples *t*-test for continuous variables and the chi-square test or Fisher’s exact test for categorical variables. Trends over time were evaluated using linear regression analysis. A *p*-value of <0.05 was considered statistically significant.

## 3. Results

### 3.1. Characteristics of Patients, Indications for Antimicrobial Therapy, and Bacterial Isolates

The total number of patients during the first study period (2013–2015) was 21,974. The mean age of patients was 55.4 ± 11.3 years (18–84), and 12,305 (56%) were males. Median hospital stay was 5.5 ± 1.2, 5.4 ± 1.3, and 5.6 ± 1.2 days in 2013, 2014, and 2015, respectively. The total number of isolates on the wards was 5855. The annual distributions of isolates, patients, and hospital stays are shown in Table 1.

The main indications for antimicrobial therapy during the first study interval (2013–2015) are summarized in Table 2. The most common indications are UTI 3374 (49.2%), respiratory tract infections 1413 (20.6%), and gastrointestinal tract infections 974 (14.2%).

The most common strains isolated during the first study period (2013–2015) were *E. coli* (15.6%), *Enterococcus* spp. (14.9%) and *K. pneumoniae* (11.9%), as shown in Table 3.

**Figure 1:**
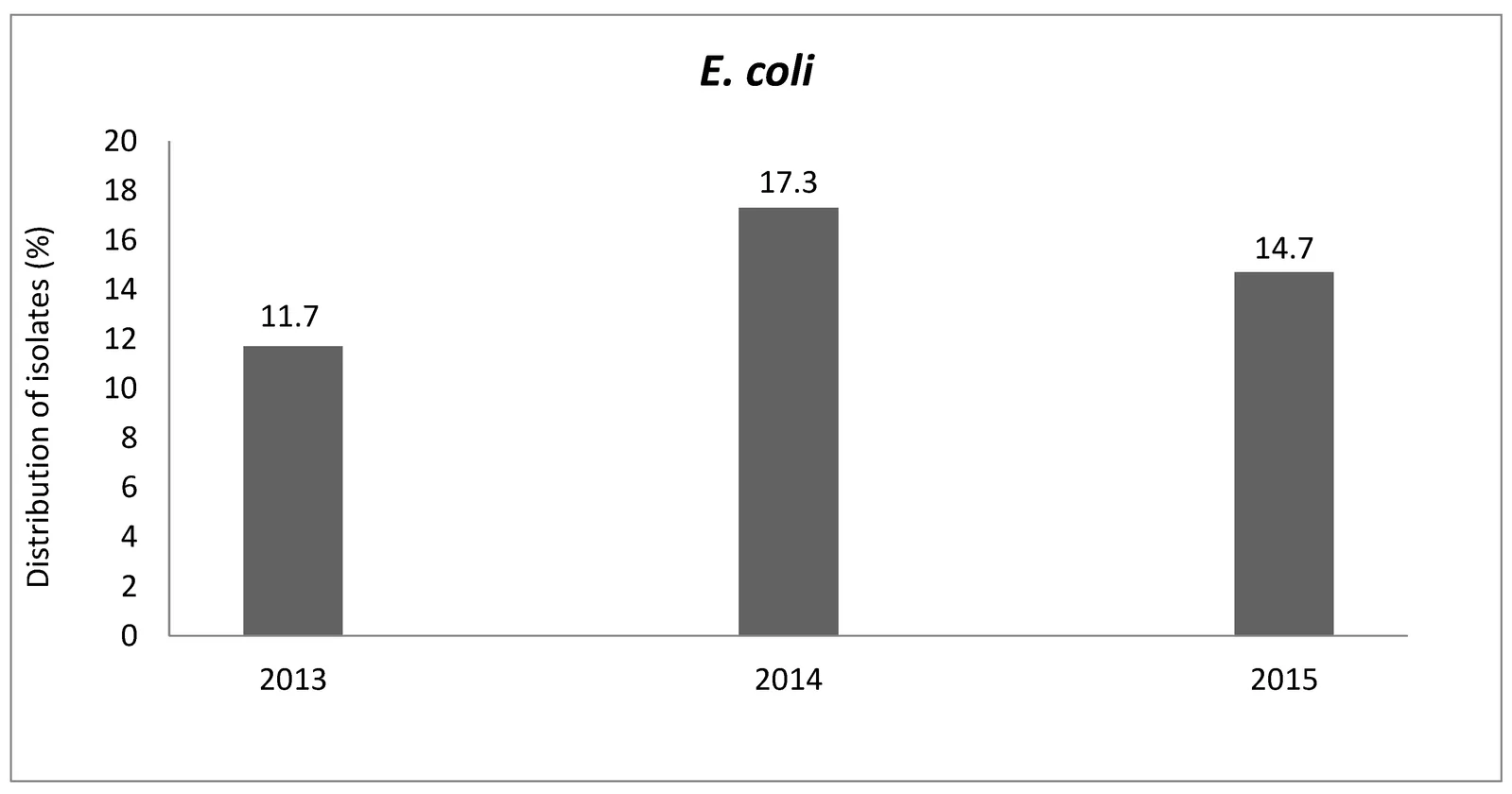
Annual distribution of *E. coli* during the first study period (2013–2015).

In Figure 1, we present the differences in the annual distribution of *E. coli* during the first study period (2013–2015). There is no statistically significant trend in the distribution of *E. coli* (in % of all isolates) during the described period (b = 1.500; *p* = 0.640).

### 3.2. Distribution of Total Number and MDR Isolates of E. coli, Antibiotic Consumption, and Profiles of Antibiotic Resistance of E. coli Isolates During 2013–2015

**Figure 2:**
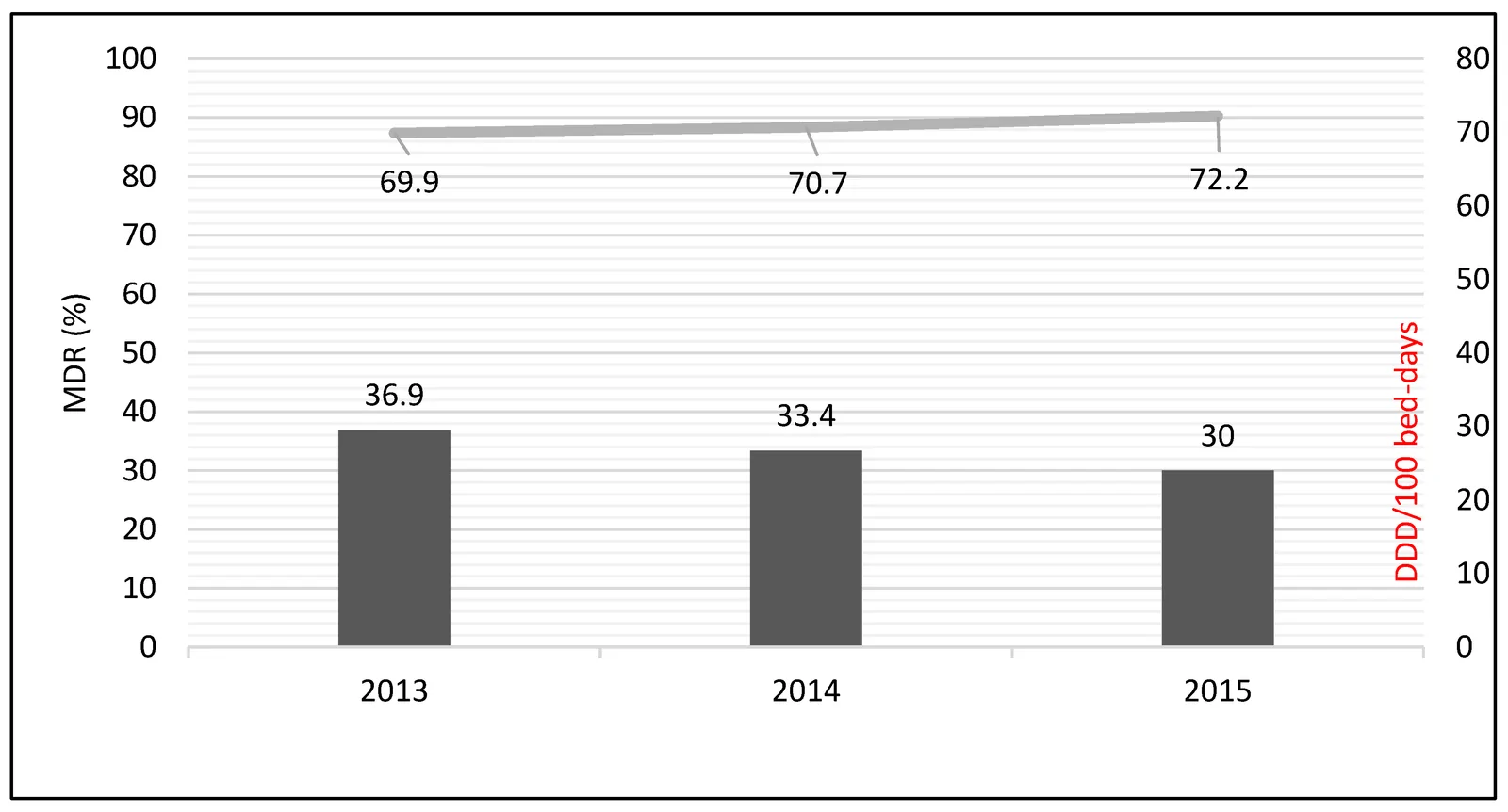
Graphical presentation of antimicrobial consumption and isolation rate of MDR *E. coli* during the first study period (2013–2015).

Occurrence of MDR *E. coli* during the whole study period was 33.2% (286/862). Linear trend analysis between the percentage of MDR *E. coli* and DDD/100 bed-days is was shown in Figure 2. The frequency of MDR *E. coli* isolates from 2013 to 2015 was 36.9%, 33.4%, and 30%, respectively. There was a statistically significant decrease in the isolation rate of MDR *E. coli* (b = −3.45; *p* = 0.005). DDD/100 bed-days were 69.9, 70.7, and 72.2, respectively. There was no statistically significant change in DDD/100 bed-days during the study period. There is no statistically significant trend in the percentage of MDR *E. coli* compared to the DDD/100 bed-days (b = 1.15; *p* = 0.111); see Figure 2 and Table 4.

The antimicrobial resistance rates of *E. coli* against the tested antibiotics during the first study period 2013–2015 and the second study period 2024 are shown in Table 5. The percentage of resistance to ampicillin significantly increased (b = 3.350, *p* = 0.038). The resistance rate of cefotaxime and cefepime decreased, but there was no statistical significance (*p* = 0.075 and *p* = 0.081). There was no significant change in the resistance rate of *E. coli* to the other tested antibiotics.

### 3.3. Distribution of Total Number of MDR E. coli Isolates, Antibiotic Consumption, and Profiles of Antibiotic Resistance of E. coli Isolates in the Second Study Period 2024

The total number of *E. coli* isolates was 479, and the total number of MDR *E. coli* isolates was 116 (24.3%). The DDD/100 bed-days during this period was 66.7. When comparing 2015 and 2024, resistance significantly increased for amoxicillin–clavulanic acid (33.4% vs. 48.2%, *p* < 0.001), cefotaxime (7.7% vs. 22.2%), ciprofloxacin (29.5% vs. 39.8%), levofloxacin (29.5% vs. 40.2%), cefepime (8.4% vs. 18.3%), ceftazidime (12.6% vs. 17.9%) and ampicillin (64.7 vs. 68.2). In contrast, resistance to ceftriaxone decreased significantly (29.5% vs. 18.5%), while resistance to trimethoprim–sulfamethoxazole remained stable (38.5% vs. 37.2%). Several new antibiotics became available during 2024 that were not available during the 2013–2015 study period, and resistance rates of *E. coli* were as follows: tigecycline 11.1%, colistin 4.9%, fosfomycin 7.5%, ceftazidim/avibactam 11.1%, and ceftalozane/tazobaktam 11.1%. The resistance rate of ceftriaxone further decreased (29.5% vs. 18.5%). See Table 5.

In the same study period, we did PCR tests in 165 septic patients. Out of the 17 samples (10.3%) where *E. coli* was confirmed in blood culture, the CTX-M resistance gene was present in 9 isolates (53%).

## 4. Discussion

### 4.1. Study Periods and Confounders

Our study design compares two distinct periods (2013–2015 and 2024) separated by an 8-year gap. The earlier period represents a historical baseline prior to full implementation of local stewardship and infection prevention measures, while 2024 reflects current practice after the introduction of molecular diagnostics. The interval reflects data availability rather than a planned interruption. Between 2015 and 2024, substantial changes occurred in infrastructure, staffing, diagnostic practices, infection control, and antimicrobial prescribing, including adjustments related to the COVID-19 pandemic. These contextual changes are acknowledged as potential confounders, and the 2024 dataset should not be interpreted as a simple continuation of the pre-pandemic epidemiological trajectory.

Interestingly, the results of our study have shown that the prevalence of MDR *E. coli* decreased during the study period from 2013 to 2015, and even further in 2024. In contrast, we previously published that the prevalence of MDR *K. pneumoniae* and *A. baumannii* significantly increased during the same time interval, in the same hospital [9].

Patients with UTI (49.2%), respiratory tract (20.6%), and gastrointestinal tract (14.2%) were most often treated. Bacterial sepsis was the fourth most frequent reason for antibiotic therapy (8.7%). This corresponds to the research of Gopaul et al. 2023, where the frequency of UTI was 42.2% [12]. In contrast, Magill et al., in a study from the USA, found a difference between 2011 and 2015 and showed that pneumonia was first, followed by gastrointestinal infections (*Clostridium difficile*), SSIs, and then UTI [13]. *E. coli* was the most frequently isolated Gram-negative bacterium in blood cultures, and it was the most common cause of UTI in European countries [14]. Additionally, *E. coli* obtained from blood cultures is the second most prevalent pathogen in our area, with *K. pneumoniae* ranking first [15]. In our study, *E. coli* was the most frequently isolated bacterium (15.7% of all isolates, ranging from 11.7% to 18.3% during the observed period). Second was *K. pneumoniae* (10.2%), which is consistent with the results of Veličković-Radovanović et al. (2015) obtained in a tertiary health institution in Niš, Serbia, during 2005–2013 [16]. Our results are consistent with the results obtained in the same hospital, where the frequency of *E. coli* in 2019 was 18.2% [17]. In another study, conducted in the Clinic for Infectious and Tropical Diseases, Belgrade (Serbia) between 2010 and 2015, *E. coli* and *K. pneumoniae* represented half of all isolates [18]. Data from Turkey indicate similar findings, where the most frequently isolated bacteria were *E. coli,* which is the number one cause of hospital infections [19]. Tasbakan et al. also show that *E. coli* was the first cause of hospital infections (45.5%), which is consistent with our data [20].

### 4.2. MDR Prevalence vs. Resistance to Specific Agents

The prevalence of MDR *E. coli* decreased during the observed period from 36.9% in 2013 to 30% in 2015, and even further in 2024–2025 (24.3%). Similarly, Gopaul et al. reported that the prevalence of MDR *E. coli* among hospital-acquired bacteremia isolates declined from approximately 27% in 2016 to 15% [12]. More than half (≈53.5%) of invasive *E. coli* isolates reported to EARS-Net in 2023 were resistant to at least one antimicrobial group under surveillance. Resistance (including fluoroquinolones and third-generation cephalosporins) is generally lower in northern Europe and higher in southern and eastern parts of the EU. Unfortunately, the resistance of *E. coli* to aminopenicillins, third- and fourth-generation cephalosporins, and fluoroquinolones in our hospital is significantly higher than in northern European countries and Great Britain [14], but lower than in countries in the Western Balkans [2]. WHO GLASS and global analyses report high median levels of third-generation cephalosporin-resistant *E. coli* (~42%), highlighting that ESBL/3GC resistance is a major contributor to MDR *E. coli* rates worldwide [2].

It is obvious that *E. coli* resistance to antibiotics is on the rise across the EU, in spite of large country-to-country differences. Similar to our study, Veličković et al. (2022) showed an increase in *E. coli* susceptibility to ceftriaxone [8]. Increasing resistance of invasive *E. coli* isolates to amoxicillin–clavulanic acid has also been reported by the European Antimicrobial Resistance Surveillance Network (EARS-Net), particularly in Southern and Southeastern Europe [21]. Spain reported a marked increase in amoxicillin–clavulanic acid resistance among bloodstream *E. coli* isolates, rising from 9.3% in 2003 to 25.3% in 2012. Also, the increase in ampicillin resistance during the period 2013–2015 and in 2024 corresponds to data reported for other European countries [21]. The observed trend for ampicillin in our study is comparable to findings from a research group in Costa Rica, which showed that *E. coli* resistance to ampicillin increased to 57% [22]. According to Mijović et al. (2020), *E. coli* resistance to aminopenicillins, ceftriaxone, and ceftazidime in a hospital in Montenegro was higher (89.06%, 70.15%, and 61.54%, respectively) than in our institution [15]. Several studies have demonstrated that recently introduced antimicrobial agents, including ceftazidime-avibactam, ceftolozane–tazobactam, fosfomycin, and tigecycline, remain highly active against MDR *E. coli*. Similar to our findings, susceptibility rates generally exceed 85–95% among *Enterobacterales*, with resistance mainly associated with carbapenemase-producing isolates [23,24,25]. In our study, colistin also retained good in vitro activity against MDR *E. coli*, with a resistance rate of only 4.9%. This finding is consistent with reports from Europe and other regions showing that colistin resistance among *E. coli* remains relatively uncommon despite the global spread of multidrug-resistant strains. A recent systematic review and meta-analysis reported a pooled prevalence of colistin resistance of 3.44% among *E. coli* isolates, which is comparable to the resistance observed in our study [26]. Likewise, a large study of bloodstream *E. coli* isolates from the Netherlands demonstrated a very low prevalence of confirmed colistin resistance (<1%), indicating that resistance remains sporadic among invasive isolates [27]. CTX-M *E. coli* are strains of the bacterium *E. coli* that produce CTX-M enzymes, a type of extended-spectrum beta-lactamase (ESBL) that provides resistance to broad-spectrum cephalosporins and penicillins. The emergence of CTX-M-producing *E. coli* has led to an expansion in community-acquired infections, such as UTIs and bloodstream infections, creating a significant challenge for treatment and requiring careful antibiotic management [28]. Previously, Hayakawa et al. reported that CTX-M *E. coli* strains were more resistant to multiple antibiotics than non-CTX-M *E. coli* strains [29]. Septic patients with risk factors for CTX-M *E. coli* isolation must be empirically treated with appropriate antibiotics. In our research, we showed that the frequency of the CTX-M resistance gene in *E. coli* in blood culture samples was 53% (9/17). In premature children from two other hospitals in Belgrade (Serbia), 33% of ESBL-producing *E. coli* produced bacterial colonization [30].

During the study time interval, 2013–2015, there was a non-significant difference in antibiotic consumption (from 69.9 to 72.2 DDD/100 bed-days). In addition, we noticed a non-significant decrease during 2024 (67.7 DDD/100 bed-days). We believe that this reduction resulted from the implementation of different measures aimed at limiting antibiotic consumption, improving hygiene in our hospital, etc. (see Method, Section 2.1). Our reported hospital usage for DDD/100 bed-days is higher than the averages in Croatia (~45.3–51.6) and Switzerland (~53), and higher than in the Turkish hospital (~55.1) [31,32,33]. Another hospital from Belgrade (Serbia), in the period 2008–2012, reported similar total antibiotic consumption (varied from 62.3 to 65.6 DDD/100 bed-days) as in our study [17]. Similarly, Peric et al. (2022) analyzed the total antibiotic utilization in another tertiary hospital in Serbia in the period from 2011 to 2021, and found an increase in DDD/100 bed-days from 38.6 in 2011 to 56.4 in 2021 [34].

A key finding is the apparent discrepancy between decreasing MDR prevalence and increasing resistance to several clinically important antimicrobials. This highlights that a reduction in the proportion of isolates classified as MDR does not necessarily indicate an overall improvement in resistance. MDR was defined as non-susceptibility to at least one agent in ≥3 antimicrobial categories, but the composition of tested agents was not fully identical across periods, as several new antibiotics became available only in 2024. Differences in tested agents, isolate composition, and testing practices may have influenced MDR classification. Thus, the decline in MDR prevalence should be interpreted cautiously and not equated with a uniform improvement in resistance patterns.

### 4.3. Cephalosporin Resistance Pattern

We observed a divergent trend in cephalosporin resistance: ceftriaxone resistance decreased from 29.5% in 2015 to 18.5% in 2024, while resistance to cefotaxime, ceftazidime, and cefepime increased. This pattern should not necessarily be interpreted as contradictory, as susceptibility to individual cephalosporins may differ substantially among *E. coli* isolates depending on the underlying β-lactamase genotype and its phenotypic expression. These findings indicate that resistance to individual cephalosporins does not necessarily change in parallel and that temporal changes in the local distribution of ESBL enzymes, particularly CTX-M variants, as well as differences in MIC distributions and susceptibility breakpoints, may contribute to the observed pattern. In addition, shifts in the composition of the hospital *E. coli* population, including changes in the prevalence of ESBL-producing clones or other β-lactamase mechanisms, may alter resistance rates for individual cephalosporins independently. Therefore, the decrease in ceftriaxone resistance observed in our study, occurring alongside increased resistance to cefotaxime, ceftazidime and cefepime, may reflect changes in the underlying resistance phenotype rather than a uniform change in overall cephalosporin resistance. This interpretation is supported by surveillance studies demonstrating that resistance trends among individual cephalosporins can differ over time. For example, a multicentre analysis of hospital-onset *E. coli* in the United States evaluated cefotaxime, ceftriaxone, ceftazidime and cefepime collectively, as extended-spectrum cephalosporins and demonstrated a substantial increase in the proportion of isolates resistant to this class between 2012 and 2020, highlighting the dynamic nature of cephalosporin resistance in hospital settings [35].

Our study has several limitations. First, the retrospective design and limited number of annual data points in 2013–2015 reduce statistical power and limit inferences about associations between antibiotic consumption and MDR rates. Second, the absence of genomic sequencing or molecular typing precludes determination of whether resistance trends reflect clonal spread, independent acquisition, or introduction of distinct lineages. Third, although Vitek^®^ 2 was the primary platform, disk diffusion and microdilution were also used, and some methodological differences between periods cannot be excluded. Fourth, the composition of tested antimicrobials was not fully identical across periods, affecting MDR operationalization. Finally, the 2024 dataset represents the post-pandemic period; changes in admissions, ICU use, infection control, prescribing, and diagnostic practices may all have influenced the epidemiology of resistant organisms. These factors should be considered when interpreting the observed changes in *E. coli* resistance patterns. However, as other authors suggested, the detection of even a single isolate of relevance can yield a much deeper understanding of the resistance drivers and epidemiological trends of pathogens or mobile genetic elements throughout the world; thus, we decided to include these data in our study [36].

The novelty of this study lies in demonstrating, based on previously published data, that resistance rates in *A. baumannii* and *K. pneumoniae* continue to rise, whereas the resistance rates of *E. coli* show a decreasing trend during certain periods [10]. This highlights that different bacterial species may exhibit distinct resistance dynamics within the same hospital environment. Additionally, we show that *E. coli* resistance during 2024 was markedly high in the subgroup of patients with *E. coli*-associated sepsis. These findings may help improve our understanding of why some patients experience prolonged hospital stay due to recurrent sepsis episodes, and they underscore the need for further targeted investigation.

At the local level, our findings provide a critical evidence base to guide empirical therapy, introduce antimicrobial stewardship programs, and support infection control interventions within our hospital. At the global level, data on *E. coli* resistance patterns from underrepresented regions remain scarce. The present study contributes to filling this gap, offering a historical baseline that complements international surveillance efforts and facilitates cross-regional comparisons of antimicrobial resistance trends.

## 5. Conclusions

This study demonstrates that antimicrobial resistance of *E. coli* is a continuously evolving process, with significant changes in resistance profiles observed over the study period. Although the prevalence of MDR *E. coli* and total antibiotic consumption remained stable or showed a decreasing trend, the comparison between 2013–2015 and 2024 revealed important shifts in antimicrobial susceptibility patterns. A significant increase in resistance to several key antimicrobial agents, including amoxicillin–clavulanic acid, ampicillin, third- and fourth-generation cephalosporins, and fluoroquinolones, indicates a progressive loss of efficacy of frequently used therapeutic options. In contrast, the continued activity of selected last-resort and newer antimicrobial agents provides valuable treatment alternatives for MDR infections, but their preservation requires careful stewardship. The detection of the blaCTX-M resistance gene among bloodstream *E. coli* isolates further confirms the dissemination of ESBL-producing strains within the hospital environment.

Continuous surveillance integrating antimicrobial consumption, phenotypic resistance, and molecular epidemiology is essential to minimize the emergence of antimicrobial resistance as an adverse consequence of antibiotic exposure.

## Figures and Tables

**Table 1:** Annual distribution of the total number of bacterial isolates and patients on the hospital wards.

Variables	2013	2014	2015
No. of total isolates	1904	1989	1962
No. of inpatients	7469	7136	7369
Mean days of hospital stay	5.5 ± 1.2	5.4 ± 1.3	5.6 ± 1.2

**Table 2:** Main indications for antimicrobial therapy for the first study period (2013–2015).

Indications	No. (%)
Urinary tract infections (UTI)	3374 (49.2%)
Respiratory tract infections	1413 (20.6%)
Gastrointestinal tract infections	974 (14.2)
Sepsis/bacteremia	598 (8.7)
Skin and soft tissue infections	123 (1.8)
Fever of unknown origin	227 (3.3)
Infection *C. difficile*	148 (2.1)
Total	6857 (100)

**Table 3:** Types and frequency of appearance of bacterial strains during the first study period (2013–2015).

Isolates	No. (%)
*Escherichia coli*	913 (15.6)
*Klebsiella pneumoniae*	697 (11.9)
*Proteus mirabilis*	457 (7.8)
*Pseudomonas aeruginosa*	445 (7.6)
*Acinetobacter baumanii*	287 (4.9)
*Enterobacter* spp.	217 (3.7)
*Staphylococcus aureus*	398 (6.8)
MRSA	111 (1.9)
*Staphylococcus epidermidis*	340 (5.8)
*Enterococcus* spp.	872 (14.9)
VRE	70 (1.2)
other	1048 (17.9)
Total	5855 (100)

**Table 4:** Total number of all isolates, *E. coli*, MDR *E. coli*, and DDD/100 bed-days during the first study period (2013–2015).

	2013	2014	2015	∑
No. isolates total	1904	1989	1962	5855
No. *E. coli* isolates	222	344	296	862
No. MDR *E. coli*	82	115	89	286
DDD/100 bed-days	69.9	70.7	72.2	

**Table 5:** Resistance rate (%) of *E. coli* during the first study period (2013–2015) and the second study period 2024.

	2013	2014	2015	2024	2013/2015	2015/2024
Antibiotic	Resistance%	Resistance%	Resistance%	Resistance%	*p*	*p*
Ampicillin	58	61.7	64.7	68.2	0.003	0.011
Amoxicillin–clavulanic acid	38	20	33.4	48.2	0.842	<0.0001
Ceftriaxone	22	22.9	29.5	18.5	0.263	<0.0001
Ceftazidime	18	17.2	12.6	17.9	0.246	<0.0001
Cefotaxime	18	11.8	7.7	22.2	0.075	<0.0001
Cefepime	12	9.8	8.4	18.3	0.081	<0.0001
Imipenem	2	3.1	1	2.5	0.684	0.0002
Meropenem	2	4.3	0	3.2	0.692	<0.0001
Gentamicin	18	17.5	19.7	15.6	0.472	0.0002
Amikacin	5	3.8	8	3.8	0.512	<0.0001
Ciprofloksacin	32	27.4	29.5	39.8	0.635	<0.0001
Levofloksacin	22	27.1	29.5	40.2	0.130	<0.0001
Trimethoprim–sulfamethoxazole	38	37.3	38.5	37.2	0.728	0.36

## Data Availability

The data that support the findings of this study are available from the corresponding author [V.Z.] upon reasonable request.

## Abbreviations

The following abbreviations are used in this manuscript:

*A. baumanni*	*Acinetobacter baumanni*
AMR	Antimicrobial resistance
ATC/DDD	Anatomical Therapeutic Chemical/Defined Daily Dose
CAESAR	Central Asian and Eastern European Surveillance of Antimicrobial Resistance
CAUTI	catheter-associated urinary tract infection
CDC	The Centers for Disease Control and Prevention
CTX-M	preferential hydrolytic activity against cefotaxime (CTX, as its acronym, -M from Munich)
*E. coli*	*Escherichia coli*
ESBL	extended-spectrum beta-lactamase
HAIs	Healthcare-associated infections
ICU	intensive care unit
*K. pneumoniae*	*Klebsiella pneumoniae*
MDR	Multidrug resistant
MRSA	Methicillin-resistant *Staphylococcus aureus*
*P. aeruginosa*	*Pseudomonas aeruginosa*
PCR	polymerase chain reaction
SSI	surgical site infection
VAP	ventilator-associated pneumonia
WHO	World Health Organization
